# Microwave-Induced Processing of Free-Standing 3D Printouts: An Effortless Route to High-Redox Kinetics in Electroanalysis

**DOI:** 10.3390/ma17122833

**Published:** 2024-06-10

**Authors:** Kornelia Kozłowska, Mateusz Cieślik, Adrian Koterwa, Krzysztof Formela, Jacek Ryl, Paweł Niedziałkowski

**Affiliations:** 1 Department of Analytical Chemistry, Faculty of Chemistry, University of Gdańsk, Wita Stwosza 63, 80-308 Gdansk, Poland; kornelia.kozlowska@phdstud.ug.edu.pl (K.K.); mateusz.cieslik@ug.edu.pl (M.C.); adrian.koterwa@ug.edu.pl (A.K.); 2 Institute of Nanotechnology and Materials Engineering, Faculty of Applied Physics and Mathematics, Gdańsk University of Technology, Gabriela Narutowicza 11/12, 80-233 Gdansk, Poland; 3 Department of Polymer Technology, Faculty of Chemistry, Gdańsk University of Technology, Gabriela Narutowicza 11/12, 80-233 Gdansk, Poland; krzysztof.formela@pg.edu.pl; 4 Advanced Materials Center, Gdańsk University of Technology, Gabriela Narutowicza 11/12, 80-233 Gdansk, Poland

**Keywords:** kitchen microwave oven, paracetamol detection, 3D printing, electroanalysis, polymer-based composites

## Abstract

3D-printable composites have become an attractive option used for the design and manufacture of electrochemical sensors. However, to ensure proper charge-transfer kinetics at the electrode/electrolyte interface, activation is often required, with this step consisting of polymer removal to reveal the conductive nanofiller. In this work, we present a novel effective method for the activation of composites consisting of poly(lactic acid) filled with carbon black (CB-PLA) using microwave radiation. A microwave synthesizer used in chemical laboratories (CEM, Matthews, NC, USA) was used for this purpose, establishing that the appropriate activation time for CB-PLA electrodes is 15 min at 70 °C with a microwave power of 100 W. However, the usefulness of an 80 W kitchen microwave oven is also presented for the first time and discussed as a more sustainable approach to CB-PLA electrode activation. It has been established that 10 min in a kitchen microwave oven is adequate to activate the electrode. The electrochemical properties of the microwave-activated electrodes were determined by electrochemical techniques, and their topography was characterized using scanning electron microscopy (SEM), Raman spectroscopy, and contact-angle measurements. This study confirms that during microwave activation, PLAs decompose to uncover the conductive carbon-black filler. We deliver a proof-of-concept of the utility of kitchen microwave-oven activation of a 3D-printed, free-standing electrochemical cell (FSEC) in paracetamol electroanalysis in aqueous electrolyte solution. We established satisfactory limits of linearity for paracetamol detection using voltammetry, ranging from 1.9 μM to 1 mM, with a detection limit (LOD) of 1.31 μM.

## 1. Introduction

Over recent years, the use of three-dimensional (3D) printing has been growing tremendously, both in science and commercial applications, mainly due to the increasing availability of printing materials and applications that make it possible to design any model [1]. 3D printing has been widely implemented in many fields, including chemistry, biology, medicine, engineering, and industry [2,3,4]. The use of novel materials for 3D printing is constantly expanding, reducing the cost of prototypes, decreasing the time needed to produce them, and creating the possibility of testing innovative types of materials in many areas of science [5,6,7,8].

Material extrusion (ME) is a technique used in additive manufacturing that is now gaining tremendous attention in technological processes and commercial applications [9,10,11]. ME is an additive technology in which the material in the form of a filament wound on a spool is fed through the extruder to a pre-heated print head, where it changes to a semi-liquid state. Then, the filament is extruded through the nozzle and distributed on the build platform, layer by layer, in three axes (x, y, z) until the full dimensions of the printed element are obtained.

PLA is one of the most popular thermoplastic polymers used in ME. Due to its origin and decomposition patterns, it has been classified as a biodegradable, compostable green polymer [12] and is thus considered a sustainable material. Filling PLA with conductive nanoparticles beyond a certain percolation threshold (typically 15% [13]) allows for 3D-printable conductive composites to be obtained. The manufacturing cost of a single electrode using this technology is very low (0.05 USD), providing an attractive alternative to other electrodes typically used in the electrochemical analysis, including the commercially available Proto-Pasta^®^ [14,15,16,17,18,19] and Black Magic 3D^®^ electrodes [20,21,22].

To ensure efficient charge-transfer kinetics, an activation process should be performed to dissolve the PLA and thus effectively enhance the conductive nanoparticle exposure of the electrolyte solution. Activation affects surface topography, increasing electrode porosity and improving the electrochemically active surface area and rate constants [23]. The simplest activation procedure described in the literature involves polishing on sandpaper to expose the graphene fragments [24]. Nevertheless, the most popular method of activating PLA-based composites for electrochemical purposes involves chemical etching by immersing the printout in an organic solvent, with the most successful results achieved using dimethylformamide (DMF) [25,26,27]. Other aprotic solvents have also been studied, but none of them has been as effective as DMF [28,29]. Other effective activation practices include hydrolysis and saponification in alkaline environments under voltametric treatment [19,30,31] and enzymatic hydrolysis with proteinase K [19,32,33]; the latter is not time-efficient. There are many other reported methods for activation of 3D-printed electrodes, including thermal annealing with a vacuum furnace at 350 °C to obtain graphitized material [34] and a wet-chemical method that involves applying ascorbic acid and sodium borohydride as reducing agents [35]. The PLA polymer matrix from 3D-printed electrodes for their activation can also be removed via complex measurement instrumentation, with methods including photo-thermal CO_2_ laser treatment [36,37], ablation with a neodymium-doped yttrium aluminum garnet (Nd:YAG) laser in air or He atmosphere [15], ablation using a femtosecond laser (FSL) [38], and cold plasma treatment with O_2_ [39].

Microwave (MW) radiation is characterized by wavelengths from 1 mm to 1 m and frequencies from 300 MHz to 300 GHz; domestic MW ovens use wavelengths of 12.2 cm, corresponding to 2.450 GHz [40,41]. The use of MW in chemistry makes it possible to carry out a reaction with or without solvent; however, in both cases, it significantly reduces the time needed for the conducted reaction. The influence of MW radiation on electrochemical processes has been investigated only for metal and carbon electrodes such as glassy carbon electrodes (GC) and boron-dopped diamond electrodes (BDD). It has been suggested that for metal electrodes, which are microwave conductors, the microwave field interacts with the solution instead of the electrode, generating high temperatures in the vicinity of the electrode. In contrast, carbon electrodes absorb microwaves, so their microwave field causes microwave heating of the carbon material [41,42]. Some reports in the scientific literature show the use of MW to modify different types of electrodes by various copper forms [43], activation of Ni_3_S_2_ nanowires on Ni foam for water electrolysis [44], activation of metal-organic frameworks [45], and electrode halogenation and oxidation of organic compounds [46,47]. However, there is no report relating to how MW influences the activation of 3D-printed CB-PLA electrodes.

In this work, MW radiation was used for electrochemical activation of CB-PLA electrodes with the hypothesis that MW induces heat sufficient for PLA removal, uncovering the conductive carbon-black filler. First, a professional microwave reactor from CEM was used to optimize the CB-PLA activation conditions under voltammetry treatment. In the next step, a kitchen microwave oven was used to verify the applicability of MW radiation using a widely available apparatus. We used this oven for the activation of all 3D-printed free-standing electrochemical cells (FSEC) in proof-of-concept paracetamol electroanalysis studies.

## 2. Materials and Methods

### 2.1. Chemicals and Instruments

All chemical compounds were used without additional purification. Paracetamol and 0.01 M phosphate-buffered saline (PBS, pH 7.4) were purchased from Sigma-Aldrich/Merck (St. Louis, MO, USA). Potassium ferricyanide K_3_Fe(CN)_6_, and potassium ferrocyanide K_4_Fe(CN)_6_ were purchased from Across Organics (Fair Lawn, NJ, USA). NaOH and KCl were obtained from P.P.H. Stanlab (Sp. z o.o., Lublin, Poland).

The MW activation of the CB-PLA electrodes was performed using the CEM Discover Microwave Synthesizer (CEM, Matthews, NC, USA) at 100 W power at different temperatures from 30 to 100 °C, applying three different treatment durations (5, 10, and 15 min). An 800 W kitchen oven with adjustable power (Sharp R270) was used in comparative studies. All the activation procedures were carried out at 10% of the power of a kitchen microwave oven (80 W) in 1 M NaOH electrolyte solution, following a different activation protocol previously published by our group [19]. To achieve the most appropriate conditions for microwave activation of CB-PLA electrodes in a kitchen microwave oven, three electrodes were activated at different times of MW exposure: 5, 10, and 15 min.

All electrochemical measurements were performed with potentiostat/galvanostat, (M204 Metrohm Autolab, Utrecht, The Netherlands) equipped with FRA32M electrochemical-impedance spectroscopy module with Nova 2.15 software. The measurements were performed in a cell consisting of the three-electrode system: for 3D-printed CB-PLA electrode (diameter of 8 mm, measurement area of 0.564 cm^2^), and FSEC (11 mm × 11 mm × 2 mm, measurement area of 1.21 cm^2^), Ag/AgCl (0.1 M NaCl) reference electrode and platinum wire as a counter electrode. A 0.01 M phosphate buffer (pH 7.4) containing 1 mM K_3_[Fe(CN)_6_] and 1 mM K_4_[Fe(CN)_6_] was used as an electrolyte solution. The cyclic voltammetry (CV) was performed in a range from −1.0 V to 1.0 V with a scan rate of 0.1 V/s. The differential pulse voltammetry (DPV) was obtained in the potential range from −0.2 V to +0.7 V, applied pulse amplitude 50 mV, potential step 10 mV, modulation time 0.07 s, and interval time 1.85 s. The electrochemical impedance spectroscopy (EIS) measurements were carried out at a frequency range from 0.1 Hz to 10 kHz. Studies were carried out at open circuit potential after 5 min of sample conditioning with a perturbation amplitude of 10 mV. All impedance data were analyzed using ZSimpWin 3.21 impedance software with the appropriately fitted electrical equivalent circuit. All EIS spectra are presented in the Nyquist plot.

SEM images were performed using an FEI Quanta 250 FEG instrument (Thermo Fisher Scientific, Waltham, MA, USA) equipped with a Schottky field-emission gun operating at an accelerating voltage of 20 kV. Raman spectra were obtained by Raman microscopy (XploRA Plus spectrometer from Horiba Scientific, Palaiseau, France). To avoid melting the investigated samples, Raman spectra were obtained by applying an argon-ion laser (514.5 nm), operating at 1% of its total power (50 mW). All contact-angle (CA) measurements were carried out at room temperature using Drop Shape Analyzer (DSA) 100, (Krüss, Hamburg, Germany) by applying 4 μL of deionized water drops to the surface of the investigated material; each measurement was taken 10 times. The analysis of the obtained data was performed using Advanced 1.7.10 by Krüss software. The analysis of the obtained results was carried out using the Young-Laplace fitting method.

### 2.2. 3D Printing Electrodes

Investigated model CB-PLA electrodes of size 11 mm × 11 mm × 2 mm were printed from commercially available Proto-Pasta^®^ electrically conductive PLA 3D printer filament 1.75 mm in diameter. The basic composition of the CB-PLA filament has been described previously [48]. The FSEC were obtained using a multimaterial print with conductive CB-PLA and non-conductive PLA filaments. The shape of the electrodes was designed by the authors and printed on the Original Prusa i3 MK3S+ 3D printer with the MMU2S multimaterial upgrade. The electrodes were printed with the following parameters: nozzle diameter—0.4 mm, nozzle temperature—220 °C, building platform temperature—60 °C, layer thickness—0.2 mm, infill—40%, extrusion multiplayer—1.1, print speed—40 mm/s, first-layer print speed—20 mm/s. The infill lines were printed at +45°/−45° degree angles. The electrochemical device was printed using Proto-Pasta^®^ filament and PLA filament (PLA silver filament made for Prusa research by Colorfil, diameter 1.75 mm). The geometry of the FSEC, which was cylindrical in shape, was as follows: 60 mm height, 22 mm radius, 11 mm × 11 mm CB-PLA located in the center.

## 3. Results and Discussion

### 3.1. The Microwave-Induced Activation of the CB-PLA Electrode

**Figure 1:**
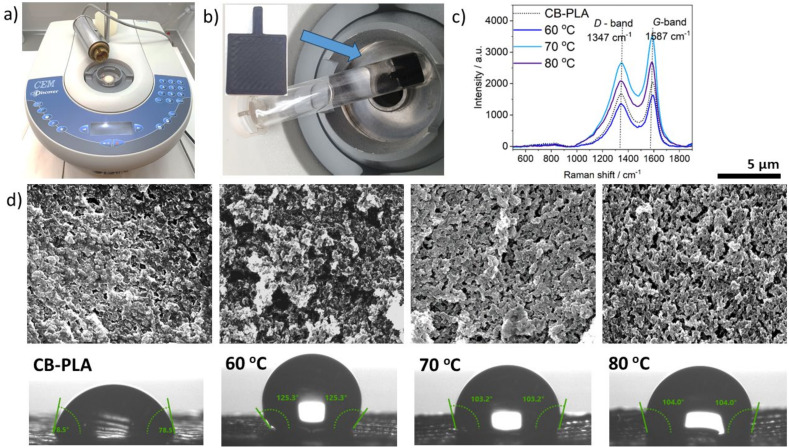
(**a**) CEM microwave synthesizer used for model CB-PLA microwave-based electrode activation, (**b**) microwave-reaction vessel and 3D-printed model CB-PLA electrode before MW activation, (**c**) Raman spectra of CB-PLA before and after microwave activation in the CEM reactor, (**d**) scanning electron microscopy (SEM) scans and contact-angle measurements for bare CB-PLA and MW-activated electrodes after 15 min microwave irradiation in the CEM synthesizer at 60 °C, 70 °C, and 80 °C.

To verify the effect of MW on CB-PLA electrode activation, a series of measurements were carried out using the CEM Discover microwave synthesizer shown in Figure 1a and model CB-PLA electrodes presented in Figure 1b. All measurements for microwave-based electrode activation were taken in dedicated reaction vessels (Figure 1b).

The most likely mechanism underlying the activation of the CB-PLA electrodes in the microwave in alkaline media is associated with the increased hydrolysis of PLA, which exposes the carbon-black layers in the electrode structure by the mechanism described previously [19]. In general, the mechanism of PLA decomposition involves hydrolysis of the ester bonds within the PLA as well as intramolecular transesterification reaction occurring at the polymer end groups. Moreover, the carbon materials including CB-PLA belong to the microwave absorbers, which behave in a manner opposite to that of the metallic electrodes, which are microwave conductors [42]. Therefore, in carbon electrodes, there is internal heating of the material [41]. The higher-intensity microwave field interacts more strongly with the carbon material than with the surrounding solution, resulting in a higher-temperature region appearing in the carbon material, including in the surrounding solution of reaction, which, in the case of CB-PLA electrodes, affects their direct activation. As the CB-PLA electrode is heated inside, the hydrolysis of PLA is induced, exposing the carbon fibers and leading to the electrochemical activation of the CB-PLA electrodes.

Electron microscopy imaging was performed to investigate the morphological changes contributing to the electrochemical activation of the model CB-PLA electrodes. Figure 1d shows SEM images for a reference CB-PLA electrode and microwave-modified electrodes at temperatures of 60 °C, 70 °C, and 80 °C. The surface structure of the reference electrode appears to be smooth; the conductive carbon-black filler is coated with a non-conductive PLA polymer. By contrast, the surface of electrodes activated by MW at 60 °C and above is different; many dimples and cavities appear on the surface of the CB-PLA electrodes, and the electrodes assume a porous morphology resembling that seen after electrochemical activation [49]. Furthermore, the conductive carbon-black filler is exposed, which affects the conductivity of the electrodes. This structure of the electrodes is created by the formation of hot spots inside the electrode and by its internal heating [41,42], which result in hydrolysis and degradation of the PLA polymer matrix. Through the comparison of morphological images of microwave-activated electrodes with electrochemical activity, it can be concluded that electrodes activated at 70 °C show the best electrochemical response, while both above and below 70 °C, the electrodes have more cracks.

The properties of 3D-printed model CB-PLA electrodes MW in a CEM microwave synthesizer were also analyzed by Raman spectra (see Figure 1c). The obtained Raman spectra for all investigated materials are characterized by two strong bands at 1347 cm^−1^ and 1587 cm^−1^. The band at 1347 cm^−1^ is associated with the D band (D denotes defects) and results from the defects in the structure of sp3 carbon materials. The band at around 1587 cm^−1^ is associated with the G band (G denotes graphitic) and indicates the graphitized carbon structure and the presence of sp^2^ hybridized carbon atoms [38,50]. The intensity ratio of the D band and G band (I_D_/I_G_) provides information about the degree of graphitization of carbon [51]. The intensity ratio (I_D_/I_G_) calculated for CB-PLA is 0.82, while those for CB-PLA electrodes microwaves activated in a CEM reactor at 60 °C, 70 °C, and 80 °C for 15 min are 0.83, 0.76, and 0.77 respectively. The lower D-band-to-G-band intensity ratio (I_D_/I_G_) improves the electrical conductivity [52] due to the increased presence of graphitic structures [50]. The lowest intensity ratio (I_D_/I_G_) for MW electrodes activated in the CEM reactor was obtained for electrodes activated at 70 °C for 15 min. This suggests that the highest conductivity associated with the presence of graphitic structures on the surface occurs in this electrode, which also demonstrates that the selection of microwave activation at 15 min appears to be the most appropriate. In addition, it can be concluded that the analysis of the above Raman spectra allows the conclusion that the greatest hydrolysis and degradation of PLA occurs at a temperature of 70 °C during the studied microwave exposure time.

The contact-angle measurements (see Figure 1d) allowed us to establish the surface properties of the studied electrodes after microwave activation during PLA degradation and exposure of the conductive carbon-black filler. The different contents of exposed carbon-black filler affect the conductivity of the electrodes. However, considering that when the carbon-black content increased to 12%, the conductivity did not change significantly [53], the microwave-induced surface roughness has a significant influence on the changes observed in the changes found in contact-angle measurements. The value of the contact angle obtained for the bare unmodified electrode was 78.5° ± 0.23°, which is also in agreement with what other authors have obtained [54,55]. After microwave activation at 60 °C for 15 min, the contact angle increases to 125.3° ± 0.64° and the surface becomes more hydrophobic. Such a significant change in the measurement of the contact account at 60 °C is attributed to changes resulting from an additional surface roughness and heterogeneity [56]. At this temperature, the hydrophobic carbon-black filler is exposed on the surface of the electrodes, which becomes rougher. The effect of surface roughness on contact-angle measurement has been described elsewhere [57]. On the other hand, microwave treatment at higher temperatures causes a decrease in the contact angle from 125.3° ± 0.64° to 103.2° ± 0.64° and 104° ± 0.49° for electrodes after microwave activation at 70 °C and 80 °C, respectively. These values are very similar to each other and to the contact angle obtained for pure carbon black, a hydrophobic material, which is 105.4° ± 2.3° [53], which means that the carbon black predominates on the surface, displacing the PLA polymer, and that the surface roughness is changed.

The main advantages of the proposed electrode-activation method using a microwave synthesizer are its short time duration (300 s to 15 min) and the possibility of applying non-toxic reagents. Compared to known other electrode-activation methods, the response time of the proposed microwave-based activation method for 3D printing is similar to that of aprotic solvent usage [25,26,27,28,29]. Moreover, the degradation of PLA with complex biomolecules is not necessary [32,33]; furthermore, in microwave activation, there is no need for sophisticated equipment like that used in the case of laser or thermal activation [15,36,37,38,39].

### 3.2. The Microwave Activation of Model CB-PLA Electrode—Electrochemical Investigations

**Figure 2:**
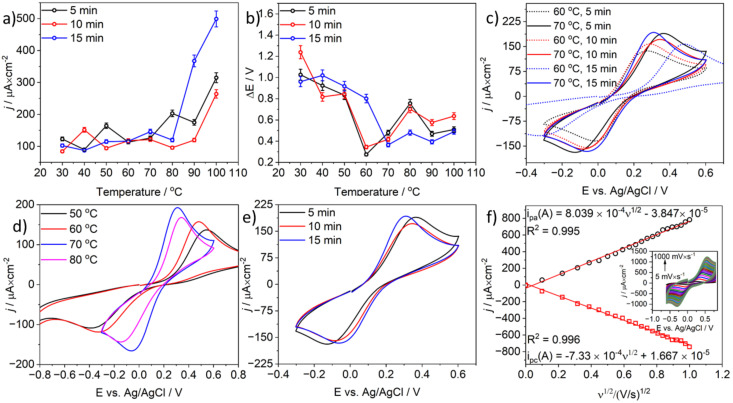
(**a**) Dependence of anodic current density of microwave-activated model CB-PLA electrodes activated in a microwave CEM reactor in a temperature range from 30 °C to 100 °C for 5, 10, and 15 min; (**b**) ΔE dependence estimated for CB-PLA electrodes microwave-activated at a temperature range of 30 °C to 100 °C for 5, 10 and 15 min. (**c**) CVs obtained in 0.01 M PBS, pH 7.4 containing 1 mM Fe(CN)63-/4 for model CB-PLA electrodes after microwave activation at 60 °C and 70 °C for 5, 10, and 15 min, (**d**) Activation for 15 min in the temperature range from 50 °C to 80 °C, (**e**) at 70 °C for 5, 10, and 15 min, (**f**) relationships between cathodic and anodic peak currents and the square root of the scan rates, in inset CVs obtained in 0.1 M KCl containing 1 mM Fe (CN)_6_^3−/4−^ at model CB-PLA electrode activated in a microwave CEM synthesizer at 70 °C for 15 min; scan rates from 0.01 V to 1 V.

The effect of MW-induced-activation on charge-transfer kinetics in the model CB-PLA electrode was studied using CV. Figure 2a shows the correlation of anodic current density calculated for all cyclic voltammograms (CVs) obtained for model CB-PLA electrodes after activation in a microwave CEM reactor in the temperature range from 30 °C to 100 °C for 5, 10, or 15 min. Nevertheless, Figure 2b shows the dependence of peak separations (ΔE) for all investigated electrodes under the aforementioned conditions. A temperature of 70 °C was found to be the most appropriate temperature for microwave activation because the anodic current density obtained for this temperature is the highest, resulting in the lowest value of peak separation (ΔE). In the case of low-temperature microwave activation conducted at 40 °C for 10 min, peaks with a high current density of 151 µA·cm^−2^ were achieved. A similar situation was observed when microwave activation was carried out at 50 °C for 5 min, where the current density was 164 µA·cm^−2^. However, the ΔE values for these electrodes were very high (817 mV and 835 mV, respectively; see Figure 1b). Furthermore, in the case of short-term microwave activation of the CB-PLA electrode, high current-density values of 203 µA·cm^−2^ were obtained after 5 min at 80 °C, but, as in the previous case, the ΔE was found to be 756 mV. It is worth noting that in the case of microwave activation conducted at high temperatures (90 °C and above), high current-density values were obtained, but the electrochemical process was inhibited, as is shown by the increasing values of ΔE parameters with temperature. Moreover, 100 °C is not a suitable activation temperature, as it leads to the degradation of the surface of the CB-PLA electrodes.

As can be seen in Figure 2b, the ΔE values depended on the temperature of the microwave activation time of CB-PLA electrodes. At low activation temperatures from 30 °C to 50 °C, the value of peak separation was very high. The greatest temperature dependence of the microwave-based activation process was observed at 60 °C. At this temperature, the lowest ΔE values (275 and 345 mV) were obtained after microwave activation for 5 min and 10 min, respectively. On the other hand, the ΔE value for microwave activation for 15 min was significantly different (ΔE 802 mV). A microwave-based activation process conducted for 15 min at 70 °C caused the greatest changes. The ΔE value decreased to 365 mV, and the current-density values for the obtained peaks were the highest at this temperature. A comparison of the CVs obtained after microwave activation of the CB-PLA electrodes at temperatures 60 °C and 70 °C is shown in Figure 2c. It is noteworthy that microwave activation carried out at 10- and 5 min led to a decrease in the current densities of the peaks with increasing ΔE (see Figure 2c). At temperatures above 80 °C, no time-dependent dependence of the microwave-based activation process was observed in the current peak density and ΔE values, which vary each time.

Figure 2d shows the CVs to illustrate the temperature effect of a 15 min microwave activation on the electrochemical activity of the investigated model CB-PLA electrodes. The lowest peak-to-peak separation value and the highest current density were achieved for electrodes activated at 70 °C. The dependence of CVs obtained from CB-PLA electrodes after MW activation at 70 °C for 5, 10, and 15 min is presented in Figure 2e, which also confirms that a temperature of 70 °C is the most appropriate temperature for microwave-based activation of model CB-PLA electrodes for further electrochemical characterization and examination of their electroanalytical properties.

The electrochemically active area of a model CB-PLA electrode microwave activated in a CEM chemical synthesizer for 15 min at 70 °C/100 W was determined using the modified Randles-Sevcik equation for a one-step, one-electron irreversible process, as in Equation (1) [58], which is given below:

i_p_ = 2.99 × 10^5^ n^3/2^α^3/2^ACD^1/2^ν^1/2^ (1)

where n is the total number of electrons transferred, α is the charge-transfer coefficient (assumed to be 0.5), n is the number of electrons involved in the charge-transfer step, A is the active surface area, C is the concentration of Fe(CN)_6_^3−/4−^, D is the diffusion coefficient for potassium ferricyanide (7.6 × 10^−6^ cm^2^s^−1^) and potassium ferrocyanide (6.3 × 10^−6^ cm^2^s^−1^) [59], and υ is the scan rate (V/s).

The active surface area was calculated using the cyclic voltammograms presented in the inset of Figure 2f obtained at different scan rates from 0.01 V/s to 1 V/s and recorded in a solution of 0.1 M KCl containing 1 mM Fe(CN)_6_^3−/4−^. The geometrical surface area of the CB-PLA electrode was 0.564 cm^2^, while the obtained electrochemically active surface areas calculated from the equations shown in Figure 2f were 0.778 cm^2^ and 0.779 cm^2^ for anodic and cathodic processes, respectively.

### 3.3. CB-PLA Electrodes MW Activation in a Kitchen Microwave Oven

In a kitchen microwave oven, only microwave power control is possible, while temperature control is impossible. We found that 80 W is the minimal wattage of a kitchen microwave oven that can be obtained. It is worth mentioning that this is one of the lowest wattages of kitchen microwave ovens (10% of 800 W).

**Figure 3:**
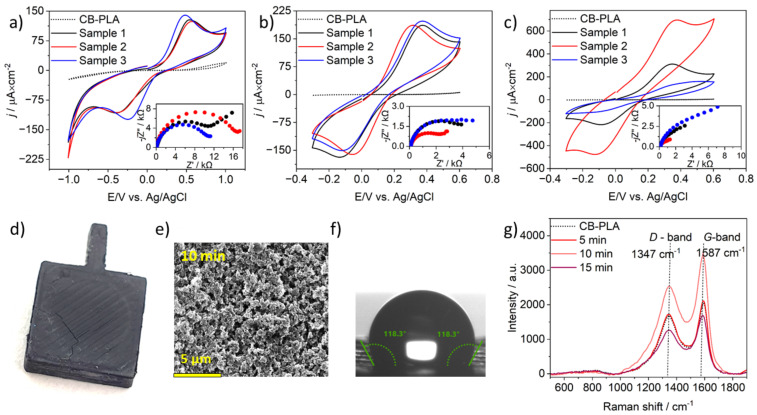
CVs and EIS spectra for a CB-PLA electrode microwave-activated in 1 M NaOH using a kitchen microwave oven for (**a**) 5 (**b**) 10 (**c**) 15 min, obtained in 0.01 M PBS containing 1 mM Fe(CN)_6_^3−/4−^, scan rate 100 mV/s. (**d**) Degradation of CB-PLA electrode after activation in a kitchen microwave oven for 15 min, (**e**) scanning electron microscopy (SEM) images of CB-PLA electrode surfaces after 10 min of microwave irradiation in a kitchen microwave oven, magnification ×10,000. (**f**) Contact-angle measurements were obtained for microwave irradiation in a kitchen microwave oven for 10 min. (**g**) Raman spectra of CB-PLA electrodes after microwave activation in a kitchen microwave oven.

The effectiveness of the microwave-based activation process in a kitchen microwave oven was verified by CV and EIS measurements with three repetitions, as shown in Figure 3a–c. The cyclic voltammograms obtained for CB-PLA electrodes after MW-induced activation for 5 and 10 min are similar. Nevertheless, the voltammograms for CB-PLA electrodes after MW-induced activation for 15 min are quite different for all three electrodes. Despite this disparity, the calculated ΔE values for these electrodes were 420 mV and 470 mV; the activation of the CB-PLA electrode in 15 min was considered inappropriate because the surface of the electrodes was usually destroyed during 15 min of activation. The exposure of CB-PLA electrodes for such a long time results in their internal heating and their decomposition [42]. The phenomenon of CB-PLA electrode degradation with a CEM synthesizer also occurs at 100 °C. CB-PLA electrodes after MW-induced activation over 15 min showed a fracture similar to that seen in those activated in a professional synthesizer CEM synthesizer at 100 °C (see Figure 3d).

The values of ΔE for CB-PLA electrodes after microwave activation for 5 min in a kitchen microwave oven are 930 mV, 910 mV, and 715 mV. Nevertheless, it is worth noting that the calculated values of ΔE for electrodes after 10 min of MW-induced activation in a kitchen microwave oven were the lowest—500 mV, 357 mV, and 481 mV—which proves that a 10-min activation time involving a kitchen microwave oven is the recommended time.

The EIS spectra are presented in Nyquist plots in the inset of Figure 3a–c for all investigated electrodes with different activation times. The R_s_(CPE(R_ct_W)) electric equivalent circuit was chosen to determine the values of the relevant electrical parameters with a good fit, as indicated by the (Chi^2^) parameter, the values of which were between 10^−3^ and 10^−5^. The selected model includes Rs (series resistance, in particular electrolyte resistance), CPE (a constant-phase element used to consider the geometric and electrical heterogeneities resulting in frequency dispersion of the capacitance effect), R_ct_ (charge-transfer resistance), and W (Warburg impedance). The calculated charge-transfer resistance values R_ct_ obtained for CB-PLA electrodes after 5 min in the kitchen microwave oven were 11 kΩ cm^2^, 15.6 kΩ cm^2^, and 10 kΩ cm^2^. The closest values of charge transfer resistance Rct, obtained after 10 min for CB-PLA electrode activation, were 3.8 kΩ cm^2^ 2.0 kΩ cm^2^, and 3.7 kΩ cm^2^. The CB-PLA electrodes with the longest exposure to microwave radiation have different values of charge-transfer resistance. The calculated Rct values of 2.5 kΩ cm^2^, 1.1 kΩ cm^2^, and 7.4 kΩ cm^2^ were obtained for the first, second, and third investigated electrodes, respectively.

The resulting R_ct_ values calculated for CB-PLA electrodes in activated a kitchen microwave oven are similar to those of electrodes electrochemically activated by anodic overpotential (about 0.6 V [19]), to those of electrodes activated by enzymatic processes, e.g., by proteinase K for 72 h, and to those of the electrodes activated by polishing and electrochemical activation [60].

Figure 3e presents the SEM image CB-PLA after microwave activation in a kitchen microwave oven for 10 min in a 1 M NaOH solution with 80 W power. The obtained images indicate that the microwave-activation process results in the development of the electrode surface in comparison with the reference electrode (see Figure 1c). The CB-PLA electrode, as mentioned earlier, under the influence of MW, heats up inside; therefore, under the increased influence of temperature, degradation of PLA occurs, the conductive filler is exposed, and the electrode surface becomes porous.

Activation in a kitchen microwave oven increases the contact angle value from 78.5° ± 0.23° to 118.3° ± 0.33°, and the surface becomes more hydrophobic (see Figure 3f). Microwave treatment in both cases degrades the PLA polymer, which exposes the carbon-black filler, increasing the hydrophobicity of the surface. This result is consistent with the results of an earlier study in the CEM synthesizer, with an effect somewhere between 60° and 70 °C, based on the CA value.

The Raman spectra presented in Figure 3g, which were obtained for electrodes activated for different times in a kitchen microwave oven, were also analyzed. As previously mentioned, the intensity ratio (I_D_/I_G_) calculated for CB-PLA was 0.82, while the intensity ratio obtained for the electrodes microwave-activated in a kitchen oven for 5 min was the same. On the other hand, for electrodes activated for 10 and 15 min, the intensity ratios (I_D_/I_G_) were 0.71 and 0.76, respectively. Since the lowest intensity ratio (I_D_/I_G_) was obtained for the CB-PLA electrode after 10 min, the formation of graphitic structures is maximized on a surface activated in this way [50]. Therefore, activation in a kitchen microwave oven confirms that the activation of CB-PLA in a kitchen microwave oven should take 10 min.

The electrochemical measurements related to CB-PLA activation of electrodes by the microwave method in a 1 M NaOH solution, employing a kitchen microwave oven, indicate that a time of 5 min is too short to achieve satisfactory electrode activation, while a treatment time of 15 min is too long to achieve satisfactory results. Meanwhile, an exposure time of 10 min for the CB-PLA electrode subjected to microwave irradiation of 80 W is suitable to activate the electrodes.

### 3.4. Paracetamol Electroanalysis with MW-Activated, CB-PLA Electrodes and Free-Standing Electrochemical Cells (FSEC)

**Figure 4:**
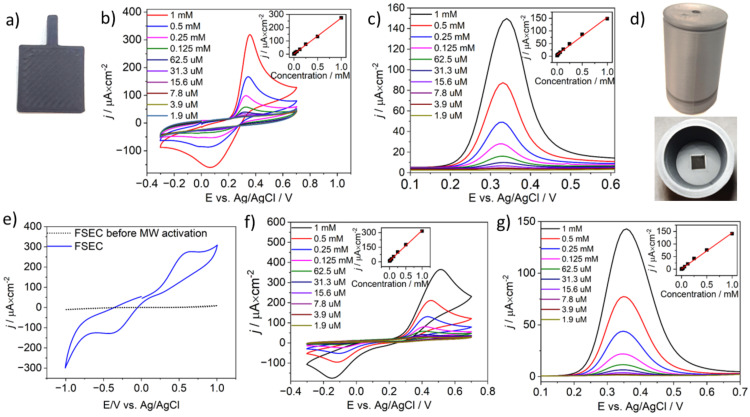
(**a**) CB-PLA electrode photograph, (**b**) cyclic voltammograms, and (**c**) differential pulse voltammograms of different paracetamol concentrations of paracetamol in 0.01 M PBS, pH 7.4 at the CB-PLA electrode activated in a microwave reactor. (**d**) FSEC photograph, (**e**) CVs of FSEC for the reference electrode and after microwave activation in 0.01 M PBS containing 1 mM Fe(CN)_6_^3−/4−^ for 10 min, scan rate 100 mV/s. (**f**) CVs and (**g**) DPVs of different paracetamol concentrations of paracetamol in 0.01 M PBS, pH 7.4 obtained for the FSEC, activated in a kitchen microwave oven.

The CB-PLA (see Figure 4a) MW-activated using a CEM synthesizer (70 °C for 15 min and 100 W) and kitchen microwave oven (80 W, 10 min) were tested as electrodes in electroanalysis of paracetamol in aqueous solution using CV and DPV techniques. The detailed mechanism of paracetamol oxidation has been described elsewhere [61].

The CVs (scan rate of 100 mV/s) in Figure 4b) show the variation in redox currents associated with paracetamol oxidation; the process should be considered electrochemically irreversible. The oxidation current reveals high linearity throughout the measurement range from 1.9 μM to 1 mM. The linear regression was given by the following equation: *j* = 0.271 C [mM] + 1.507 [μA·cm^−2^], and the correlation coefficient (R^2^) was 0.999; see inset of Figure 4b. The limit of detection (LOD) was calculated to be 1.63 μM. The LOD was estimated using the equation 3δ/slope, where δ is the standard deviation of the intercept and slope is the slope of the calibration curve [62]. Similar results were obtained by DPV, as seen in Figure 4c. A slightly lower DPV response was observed compared with CV scans, allowing us to estimate the LOD to be 3.74 μM. The calculated linear regression equation was *j* = 0.148 C [M] + 5.551 [μA·cm^−2^], and the correlation coefficient (R^2^) was 0.992.

Next, an electrochemical FSEC (photograph shown in Figure 4e) was designed to investigate the possibility of employing 3D-printing methods to obtain electrochemical devices under home conditions using a kitchen microwave oven. The 3D printing was carried out using a dual-material printing technique designing the placement of a CB-PLA electrode of dimension, which was used for microwave activation.

The electrochemical response of FSEC was investigated in a solution containing Fe(CN)_6_^3−/4−^ as a redox couple after 10 min of microwave activation, with results depicted in Figure 4e. The CV analysis proves that the electrochemical process takes place, but its reversibility is significantly hampered, as shown by a ΔE value of 950 mV. Alteration in ΔE values relate to cell-geometry-dependent differences in microwave distribution and hot-spot formation, resulting in suboptimal PLA removal.

The paracetamol-detection results obtained using an FSEC are depicted in Figure 4f,g. The paracetamol detection was performed under the same operating conditions earlier used for the CB-PLA electrode studies. Comparing the shape of voltammeters obtained under the same solutions at the same pH (0.01 M PBS, pH 7.4) in a model CB-PLA electrode activated in a CEM microwave synthesizer to the results obtained using an FSEC after microwave-induced activation in the kitchen microwave oven, it can be concluded that the latter leads to larger redox-potential splitting, which originates from the nature of electrochemical cell geometry, as discussed earlier.

Regardless of the hampered electrode kinetics, the CVs for paracetamol detection with an electrochemical cell MW-activated in a kitchen oven reveal a high linearity coefficient in the paracetamol concentration range, as shown by the linear regression equation (*j* = 0.312 C [M] + 0.103 [μA·cm^−2^] and R^2^ = 0.996). The calculated LOD was 1.31 μM. In the DPV experiment, the LOD was estimated to be 3.0 μM, and the linear regression equation was *j* = 0.143 C [M] + 1.155 [μA·cm^−2^], with R^2^ = 0.998. The dependence of oxidative peak current obtained using the CV and DPV methods shows very good linear dependence in the measurement range from 1.9 μM to 1 mM (see inset of Figure 4f,g), allowing us to conclude that while it lowers transfer kinetics, the electrochemically active surface area remains effective for paracetamol detection in the studied concentration range.

The results were compared to those obtained with other 3D-printed devices and electrodes. The effect of MW-induced activation in a kitchen microwave oven is similar (2.84 μM by DPV method) to the effect obtained using an electrochemical cell-on-a-chip device fabricated entirely by 3D printing [63,64]. Meanwhile, the use of CB-PLA filament and microwave activation allows for detection limits only one level lower (0.5 μM by the DPV method) than that which is possible for electrodes fabricated with PLA/graphene filament, which is not commercially available [64].

## 4. Conclusions

This paper presents the use of 3D printing for electrochemical research. In the first part of the research, model CB-PLA electrodes were 3D-printed and activated using a microwave reactor. We have established that the recommended activation protocol for a model CB-PLA electrode is 15 min at 70 °C using a 100 W microwave reactor. Next, our work revealed the capability of the household microwave oven for effective electrode activation. The obtained results indicate that the 10-min treatment in 1 M NaOH is sufficient to significantly enhance charge-transfer kinetics. It is also possible to 3D=print and activate a complete electrochemical cell. The FSECs were used for proof-of-concept paracetamol detection, achieving detection limits similar to these obtained with the model CB-PLA electrodes. Thus, the manuscript demonstrates the utility of 3D-printed FSECs prepared with commonly-available infrastructure and without the necessity of toxic or expensive reagents.

## Data Availability

Data are contained within the article. Additional data available on request from corresponding authors.
